# Adsorption–Degradation Integrated Approaches to Mycotoxin Removal from Food Matrices: A Comprehensive Review

**DOI:** 10.3390/toxins17110556

**Published:** 2025-11-12

**Authors:** Xiyu Yang, Mingjian Yao, Wenchao Liao, Xiaoyang Li

**Affiliations:** 1 State Key Laboratory of Food Science and Resources, School of Food Science and Technology, Nanchang University, Nanchang 330047, China; 2 College of Life Science and Technology, Beijing University of Chemical Technology, Beijing 100029, China

**Keywords:** food safety, adsorption–degradation coupled systems, mycotoxin detoxification

## Abstract

Mycotoxin contamination is a crucial issue in food safety. However, the removal of trace amounts of mycotoxins from complex food and feed matrices without significant loss of nutritional and flavor quality remains a significant challenge. The integrated adsorption–catalysis strategy involves immobilizing catalytic modules onto adsorption materials, enabling in situ degradation while enriching the mycotoxins. This approach can significantly reduce the dosage of detoxification agents and achieve efficient removal of trace mycotoxins in food. This review provides an overview of adsorbents with enrichment capabilities and their applications in the targeted removal of mycotoxins from food. The adsorption–degradation coupled systems are categorized into the following two main types: adsorption–photocatalysis coupled systems and adsorption–biocatalysis coupled systems. The review introduces recent advances in the design of bifunctional catalysts, focusing on their synergistic mechanisms and practical applications for detoxifying various mycotoxins in food matrices. Finally, the review discusses current industrial challenges and offers insights into future directions for this field.

## 1. Introduction

Mycotoxins are highly toxic secondary metabolites produced by fungi, which are major contaminants in food and agricultural commodities [1,2]. Genera such as *Aspergillus*, *Fusarium*, and *Penicillium* frequently contaminate cereals, nuts, and dried fruits, producing mycotoxins including aflatoxins (AFs), patulin (PAT), ochratoxin A (OTA), zearalenone (ZEN), deoxynivalenol (DON), and fumonisin B (FBs) [3,4,5]. These persistent contaminants permeate the entire food production chain from pre-harvest to storage and processing, causing substantial economic losses [6,7]. The Food and Agriculture Organization (FAO) estimates that about 25% of global agricultural production (roughly 1 billion metric tons) is adversely affected by fungal contamination annually [8]. In the United States, annual economic losses attributed to crop damage reach approximately USD 932 million [9]. Due to their potent carcinogenic, teratogenic, and mutagenic properties, mycotoxins pose serious health risks in chronic dietary exposure even at trace level [10,11,12,13]. To reduce the risk of mycotoxin contamination, strategies including physical (adsorption, radiation, ultrasound, etc.) [14,15], chemical (uses of oxidants and fungicides, ozone treatment, etc.) [16,17], and biological (microbial and enzymatic degradation) methods have been developed [18,19,20]. Contemporary detoxification approaches for food applications can be primarily classified into the following two categories: adsorption-based [21] and degradation-based strategies [22,23].

Adsorption approaches employ engineered nanoporous and magnetic adsorbents [24,25,26], particularly metal–organic frameworks (MOFs) and their derivatives [27], biochar [28,29], and covalent organic frameworks (COFs), that leverage high specific surface area, tunable porosity, structural flexibility, abundant active sites, and facile functionalization to optimize mycotoxin–adsorbent interactions [30,31]. Particularly, molecularly imprinted polymers (MIPs), designed with molecular-level complementarity to target toxins, provide superior binding selectivity and enhanced removal efficiency, enabling precise enrichment and source-specific mitigation [32,33]. These advanced materials are widely applied in liquid food matrices such as fruit juices, beer, and milk. The degradation approaches include cold plasma and photocatalysis, which generate reactive oxygen species (ROSs) that disrupt mycotoxin structures [34,35]. Nano-photocatalysts are engineered (including binary, ternary, and hybrid photocatalytic nanomaterials) to promote surface-mediated formation of potent oxidizing radicals for efficient toxin degradation [36,37].

However, it is a great challenge to remove trace-level mycotoxins from complex food and feed matrices while preserving their original nutritional and flavor quality. Conventional adsorption-based strategies are constrained by finite adsorption capacity, and overdosing adsorbents can compromise the nutritional and sensory qualities of foods [38,39,40]. For degradation techniques, the typically low mycotoxin level, often near regulatory limits, combined with the compositional complexity of food matrices hinder reaction efficiency and control [41,42,43]. Together, these limitations underscore a persistent challenge: developing robust, highly selective methods for the precise and efficient removal of trace mycotoxins from complex food systems.

Recent advances in hybrid nanomaterials enable in situ adsorption–degradation systems that immobilize catalytic moieties within adsorption carriers via adsorption, encapsulation, or covalent coupling strategies [44,45,46]. The adsorption–degradation integrated approaches reduce the required dosage of detoxifying agents while enabling precise, efficient mycotoxin removal from complex food matrices. The carriers of these hybrid nanomaterials serve dual critical functions. On the one hand, they create protective microenvironments that enhance catalyst specificity, stability, and durability without sacrificing activity [47,48]. On the other hand, they locally concentrate mycotoxins near active sites, significantly improving mass transfer and accelerating reaction kinetics [49,50]. Compared with monofunctional catalysts, these bifunctional adsorption–degradation hybrids achieve superior extraction and conversion of trace mycotoxins through synergistic interactions between catalysts and carriers [51,52]. Moreover, the potential application scope of the hybrid nanomaterials can be broadened by rationally engineering architectures to enable targeted molecular interactions in heterogeneous food systems.

**Figure 1:**
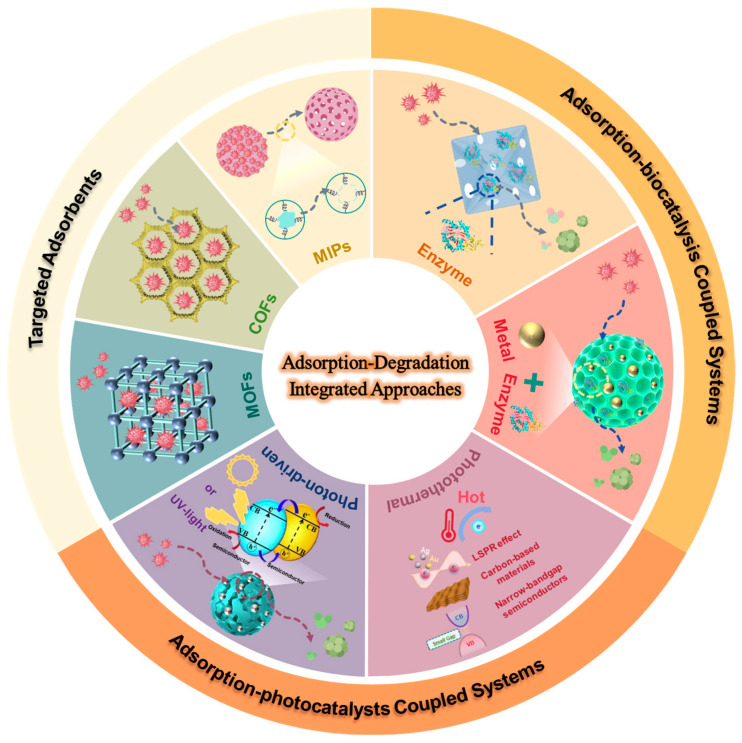
Adsorption–degradation integrated systems for mycotoxin removal in food.

This review systematically summarizes recent advances in bifunctional hybrids for mycotoxin removal in food matrices. The structural designs of targeted adsorbents such as MOFs, COFs, MIPs are summarized. The review also classifies and discusses current bifunctional catalytic systems for food applications, including adsorption–photocatalysis coupled and adsorption–biocatalysis coupled systems, focusing on the synergy mechanisms of the in situ adsorption–degradation systems for mycotoxin removal in food matrices (Figure 1). Finally, challenges to industrial implementation and future research directions are highlighted.

## 2. Targeted Adsorbents

Targeted adsorbents are a cutting-edge class of precision-engineered materials tailored to bind specific molecules through techniques such as molecular imprinting and biofunctional modification [27,53]. They feature precisely functionalized surfaces and architecturally optimized pore structures that enable highly selective capture of mycotoxins in complex matrices [26,54]. Unlike conventional adsorbents (e.g., activated carbon, silica gel, zeolites, polymeric resins, and clays), that rely largely on nonspecific interactions such as van der Waals forces and electrostatic interaction forces, targeted adsorbents achieve exceptional specificity by incorporating tailored binding cavities and bio-affinity modules (e.g., aptamers and antibodies) or rationally engineered porous frameworks [24,55,56,57]. In the adsorption–degradation coupled catalytic systems, the most widely used targeted adsorbents include MOFs, COFs, MIPs, and surface-modified derivatives of traditional adsorbents. The following sections elucidate the design strategies and interaction mechanisms by which these materials recognize and capture mycotoxins in food safety applications.

### 2.1. Metal–Organic Frameworks (MOFs)

Formed through the coordination of metal ions or clusters with organic bridging ligands, MOFs are a type of highly ordered crystalline porous materials [58]. With their exceptionally high surface areas, tunable pore architectures, and controllable topologies, MOFs can be structurally engineered to achieve selective adsorption of mycotoxins in foods [59]. Three principal strategies underpin this selectivity [24,59,60,61,62,63], they are as follows: (1) π-π stacking interactions between aromatic linkers and mycotoxin molecules, (2) introducing unsaturated metal sites (Zr^4+^, Fe^3+^, and Cu^2+^) as high-affinity coordination centers, and (3) installing functional groups (-NH_2_, -SH, and -COOH) to tailor interfacial chemistry. These strategies significantly improve the adsorption efficiency and molecular recognition specificity of MOFs by exploiting specific interactions with target mycotoxin structures, enabling highly selective capture in complex matrices.

**Figure 2:**
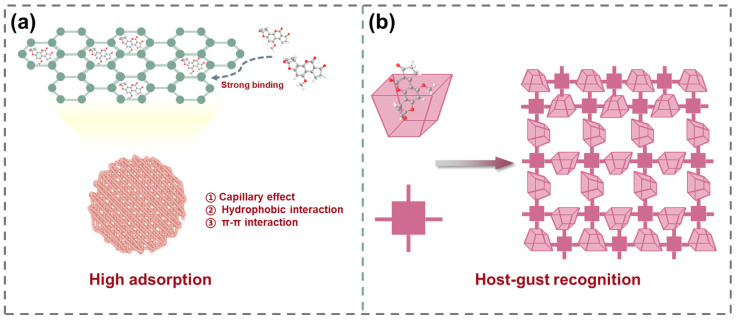
(**a**) Schematic illustration for the design and preparation of Cu-BTC MOF with high adsorption efficiency derived porous carbonaceous materials for removal of AFB_1_. (**b**) The synthetic procedure of CX4-Tph-COF and adsorption scheme of host–guest recognition with mycotoxins.

Ma et al. [64] developed a copper-based porous carbonaceous MOF (C-Cu-BTC MOF) for efficient aflatoxin B_1_ (AFB_1_) removal from vegetable oils. Using copper-based MOF as a precursor, carbonized porous derivatives were synthesized via calcination at three distinct temperatures (400 °C, 600 °C, and 800 °C). The surface-modified hybrid carbon network featuring a conjugated π-structure, provided abundant chemical binding sites for AFB_1_ while improving stability in humid environments and preserving porosity (Figure 2a). Remarkably, C-Cu-BTC MOF-600 showed optimal performance, achieving 90% AFB_1_ removal from vegetable oils within 30 min. Biosafety assessments and edible oil quality evaluations indicated substantially reduced cytotoxicity in detoxified oils, with preservation of quality attributes.

To simultaneously remove aflatoxins and zearalenone from peanut oils, Du et al. [65] synthesized an Fe-based MOF (MOF-235). The topology structure is established by basic triangular prism topological building blocks, which are formed by interconnected oxygen-centered trinuclear iron clusters with benzene dicarboxylates. Remarkably, this optimized adsorbent achieved removal efficiencies of 96.1% for aflatoxins and 83.3% for zearalenone within 30 min, while maintaining structural integrity and reusability. X-ray photoelectron spectroscopy (XPS) elucidated the underlying adsorption mechanisms, in which aflatoxins and zearalenone were adsorbed through interaction with the active site of MOF-235. The increased π–π signals of MOF-235 after adsorption of mycotoxins implicated π–π stacking between the benzene-carboxylate-modified framework and aromatic toxin rings of aflatoxin and zearalenone molecules. Additionally, decreases in the Fe 2p_3/2_ and Fe 2p_1/2_ binding energies indicated potential chelation of Fe sites with the dicarbonyl groups of mycotoxins or electrostatic interactions with the ionic dipole of the ketone carbonyl group in toxin molecules. For industrial applicability assessment, MOF-235 was packed into purification columns, demonstrating a rapid reduction in five mycotoxin contaminants from initial concentrations of 50 μg/kg to below 10.5 μg/kg. This work establishes a practical platform for engineering MOFs for the removal of multiple mycotoxins in vegetable oils. Liu et al. [66] constructed a UiO-66(NH_2_)-derived composite which distinguishably augmented active sites through strategic functionalization with cysteine and co-immobilization of gold nanoparticles (UiO-66(NH_2_)@Au-Cys) for PAT removal from apple juice. The amine, hydroxyl, and carboxyl groups from Cys enhanced the targeted binding with PAT, compensating for the shortcomings of UiO-66(NH_2_). Structural characterization via scanning electron microscopy (SEM), Fourier-transform infrared spectroscopy (FT-IR), and X-ray diffraction (XRD) confirmed the successful Au nanoparticle integration and cysteine functionalization in UiO-66(NH_2_)@Au-Cys. The composite demonstrated an adsorption capacity of 4.38 µg/mg with low cytotoxicity, which was 10 times higher than inactivated microorganisms, while reducing adsorption time from 24 h to 3 h. In a greener design, vitamin B_3_ is used as an organic linker coordinating with cobalt to prepare MOFs for extracting aflatoxins in commercial soy milk [67]. This approach achieved high adsorption capacity for aflatoxins (AFB_1_, AFB_2_, AFG_1_, and AFG_2_), with extraction recoveries of 64–75% within acceptable analytical ranges.

### 2.2. Covalent Organic Frameworks (COFs)

While MOFs offer high surface areas and tunable porosity, their practical use in foods can be limited by aqueous instability, potential metal leaching, and matrix interference [68]. In contrast, COFs are crystalline porous materials built from precisely designed organic monomers linked by robust covalent bonds, conferring superior structural integrity and chemical stability across wide pH and temperature ranges [69,70]. COFs combine ultrahigh surface areas with molecularly tunable pore architectures [71,72], and their adsorption performance can be enhanced through molecular-level design [73,74], such as installing specific functional groups and tailoring pore size/shape to optimize multiple interaction mechanisms (hydrogen bonding, π–π stacking, electrostatic interactions) [75]. The chemical diversity of COF building units also facilitates the integration of chemical and biological catalytic sites, enabling sophisticated adsorption–catalysis coupling within a single framework and addressing key limitations of conventional porous adsorbents [68,71].

Although the porosity and active sites of COFs can be engineered via building-unit and framework-network modulation, selective guest molecule recognition in food matrices imposes stringent design requirements for COFs adsorbents. To realize both host–guest recognition functionality and multiscale pore-size distribution, Xie et al. [76] incorporated dual macrocycles with defined cavities into a single framework, creating a double-macrocyclic COFs with hierarchical pores (CX4-Tph-COF) (Figure 2b). In detail, an aldehyde-functionalized macrocycle CX4-CHO was synthesized from hexamethylenetetramine. The assembly of CX4-CHO and nitrogen-rich tetrakis (4-aminophenyl) porphyrin (Tph) building units into spherical crystals was achieved through a Schiff base reaction. High-magnification transmission electron microscopy (TEM) and SEM confirmed the uniform spherical morphology of CX4-Tph-COF. Brenner–Emmett–Taylor (BET) analysis revealed a bimodal pore-size distribution. These results demonstrated the successful construction of double macrocycles with hierarchical porosity. Adsorption size-screening test and dye-adsorption studies indicated that precise size matching is pivotal for target recognition of CX4-Tph-COF. Target molecules exhibiting precise size complementarity demonstrate optimal binding affinity, driven by synergistic contributions from electrostatic interactions, hydrogen bonding, and π–π stacking, all of which collectively enhance adsorption performance. The dual-macrocyclic architecture provides abundant recognition sites, increases binding energy, and improves size-selective capture of diverse hazardous compounds in complex matrices, thereby promoting efficient and targeted enrichment of mycotoxins. For example, the maximum adsorption capacity for ZEN reached 298.6 mg/g, consistent with the model prediction and more than 30% higher than that of other reported adsorbents, which exhibits significant potential for toxin enrichment in complex food matrices.

A practical hurdle for COFs in food applications is material recovery, owing to their low density and dispersibility. To address this limitation, strategies involving magnetic functionalization and membrane fabrication have been developed. For instance, Li et al. [77] fabricated a flower-patterned COFs fiber membrane (PAN@COF FM). Firstly, polyacrylonitrile (PAN) fibers containing 2,5-divinylterephthalaldehyde (DVA) were prepared by electrospinning. Subsequently, COFs were in situ synthesized on the membrane via immersion in 3,5-tris(4-aminophenyl) benzene (TPB) solution for AFB_1_ removal. Simulations and characterization indicated that adsorption is dominated by hydrogen bonding and π-π stacking. In real water samples, the membrane maintained an AFB_1_ absorption rate above 98% after ten consecutive regeneration cycles, demonstrating excellent reusability relevant to food detoxification processes. In a complementary approach, a magnetically responsive COFs (M-COF) was developed for extracting aflatoxins (AFs) from milk, rice, and edible oils [78]. Fe_3_O_4_ coating on the COF surface formed a core–shell structure for magnetic dispersive solid-phase extraction (MDSPE), achieving extraction efficiencies ranging from 82.8% to 103.6%, while maintaining operational stability for over eight reuse cycles.

### 2.3. Molecularly Imprinted Polymers (MIPs)

MIPs have gained significant attention as a class of promising enrichment materials for mycotoxin purification. In molecular imprinting, functional monomers and cross-linkers are polymerized around a template molecule, followed by template removal which leaves three-dimensional recognition cavities whose size, shape, and functional group orientation are complementary to the target analyte [32,33]. These selective binding sites endow MIPs with high affinity and specificity for particular mycotoxins in complex matrices. However, compared to COFs, MOFs, and conventional adsorbents, MIPs suffer from several intrinsic limitations including restricted accessibility of active sites and slow mass transfer, which hinder their implementation in food systems [79]. Consequently, current research focuses on strategies such as surface imprinting and sacrificial support techniques to bring recognition sites to the exterior, increase porosity, and accelerate diffusion [80].

**Figure 3:**
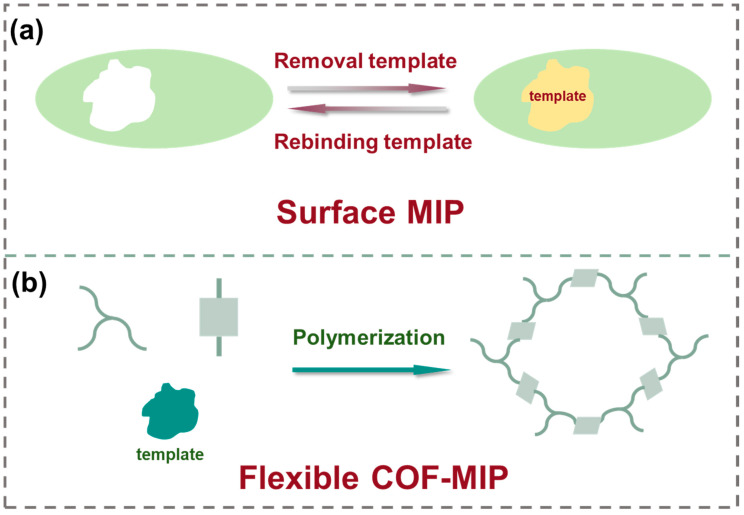
(**a**) Scheme for the fabrication of UiO-66-NH_2_@MIP. (**b**) Schematic diagram for the synthesis of MI-FCOF.

Surface-imprinted MIPs confine recognition sites exclusively at the particle interface, markedly reducing the mass transfer distance for target analytes. As illustrated in Figure 3a, a MIPs layer was grafted onto the UiO-66-NH_2_ surface to synthesize a surface-imprinted polymer (UiO-66-NH_2_@MIP), which was subsequently employed as a sorbent for aflatoxins (AFs) extraction from cereals [81]. Initiator dosage, reaction temperature, and cross-linker type were optimized to balance imprinting fidelity and site accessibility. The resulting core–shell nanocomposites formed via precipitation polymerization exhibited selective adsorption of AFs.

In a related advancement, Su et al. [82] designed a molecularly imprinted flexible covalent organic framework (MI-FCOF) for selective extraction of aflatoxins from spiked rice, corn, wheat, and peanut samples. Incorporating flexible chain segments into the porous framework increased rotational and conformational freedom, enabling dynamic cavity adjustment and stronger host–guest interactions during imprinting. The MI-FCOF was synthesized using a one-step approach involving flexible building blocks, functional monomers, and template molecules, which enabled dynamic structural transformations during the formation of COFs (Figure 3b). Therefore, the MI-FCOF exhibited strong self-adaptive abilities, making it more flexible in interacting with guest molecules and maximizing the host–guest interactions. Moreover, the combination of large surface area and accessible imprinted sites in MI-FCOF resulted in significantly enhanced adsorption capacity and selectivity for mycotoxins. Specifically, adsorption equilibrium was achieved within 3 min in a 30 mg/L aflatoxin (AFT) solution when using 1 mL of adsorbents. The maximum adsorption capacity, calculated by the Langmuir model, reached 258.4 mg/g, which is three times higher than that of the non-imprinted FCOF (NI-FCOF). Selectivity studies confirmed superior affinity for AFs over other mycotoxins.

Anene et al. [83] fabricated an MIP thin film on silica supports for PAT adsorption. The silica was prefunctionalized with surface polymerizable groups via co-condensation of tetraethoxysilane (TEOS) and γ-methacryloxypropyltrimethoxysilane (γ-MPTS), enabling subsequent MIP grafting using PAT as the template. The MIP films exhibited uptake capacities which were four times higher than those of the corresponding non-imprinted materials (NIPs) and achieved complete adsorption saturation within 20 min. These findings provide a theoretical foundation and practical basis for the application of MIPs in the efficient removal of mycotoxins from food products.

## 3. Adsorption–Photocatalysis Coupled Systems

Photocatalysis, which utilizes renewable light sources to drive targeted chemical reactions, has emerged as a widely adopted approach for water pollution control and air purification and is increasingly explored for food detoxification [84,85]. The photocatalytic detoxification process involves the following critical steps [37]: When a solid photocatalyst absorbs photons (hν) with energy equal to or greater than its bandgap (Eg), electrons (e^−^) are promoted from the valence band (VB) to the conduction band (CB) while generating holes (h^+^) in the VB. The photogenerated charge carriers then migrate to the catalyst surface, where the reductive electrons facilitate the formation of superoxide anions (·O_2_^−^) and the oxidative holes oxidize water or hydroxide to generate hydroxyl radicals (·OH). These reactive oxygen species (ROSs) subsequently initiate oxidative degradation of mycotoxins in aqueous solutions, ultimately mineralizing them into small molecules such as carbon dioxide (CO_2_) and water (H_2_O).

Due to environmental friendliness, minimal secondary pollution, and cost-effectiveness, photocatalysis shows great potential for mycotoxin degradation in food systems [86,87]. The catalytic performance of photocatalysts can be enhanced by increasing active site density, intensifying light absorption, accelerating charge separation, suppressing electron–hole recombination, facilitating charge transport, and optimizing interfacial reaction pathways [88,89]. However, the photogenerated reactive radicals, which play pivotal roles in breaking down mycotoxins, are easily quenched during mass transfer from the heterogeneous photocatalyst to the reaction system. In addition, compared with purely aqueous applications, implementing photocatalysts in foods necessitates careful attention to safety, light absorption/penetration, and matrix compatibility [90,91,92].

Combining the adsorption function with photocatalysis to construct a system with dual functions, which can concentrate toxins at the active sites and activate multiple synergistic mechanisms, is an important approach for achieving precise photocatalytic removal of mycotoxins in food matrix [93]. Based on the mechanisms of light energy utilization employed in food systems, adsorption–photocatalysis synergistic systems can be distinctly classified into the following two primary categories: (1) purely photon-driven systems encompassing ultraviolet-light-activated and visible-light-activated systems, and (2) photothermal-assisted catalysis systems.

### 3.1. Purely Photon-Driven Systems

#### 3.1.1. Ultraviolet-Light-Activated Systems

Ultraviolet (UV) light, with its typically high photon energy, can directly decompose a wide range of recalcitrant organic contaminants and toxic substances by generating highly reactive oxygen species [94]. Particularly, for wide-bandgap semiconductors such as TiO_2_, UV irradiation ensures strong oxidative performance [94,95]. Xu et al. [96] synthesized rGO-doped TiO_2_ via hydrothermal treatment (HT-rGO/TiO_2_) to degrade DON in beer. LC-MS analysis revealed that the detoxification of DON resulted from the disruption of key structural motifs in DON, including the epoxy group at C12-C13, the hydroxyl at C3, and the C9–C10 double bond.

The unique organic–inorganic hybrid nature of MOFs promotes spatial separation of photoinduced charge carriers [97]. Their inherent substrate enrichment capability enhances photocatalytic degradation efficiency through synergistic effects. Furthermore, incorporating magnetic nanoparticles addresses the critical challenge of recovering heterogeneous photocatalysts, such as graphene oxide-based composites, from complex food matrices. For example, Samuel et al. [98] designed a recyclable GO/Cu_3_(BTC)_2_/Fe_3_O_4_ hybrid nanocomposite that achieved 99% of AFB_1_ removal under UV irradiation, demonstrating a versatile approach for food-applicable photocatalytic composites.

**Figure 4:**
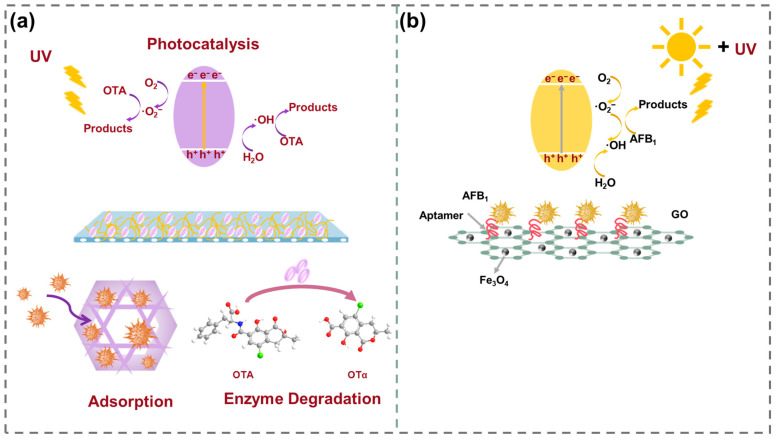
(**a**) Synergistic scheme for the removal of OTA from edible oil of PCN-222(Mn)/biomembrane composite under visible-light. (**b**) Schematic illustration of the photocatalytic mechanism of AFB_1_ by MGO/TiO_2_-aptamer photocatalyst under UV light.3.2. Photothermal-assisted catalysis systems.

Featuring a tri-modal synergistic mechanism combining adsorption, nanozyme catalysis, and photocatalysis, Zhu et al. [93] developed a zirconium-based MOFs for OTA removal in edible oils. Utilizing the high toxin-adsorption surface area (172.29 m^2^/g) of PCN-222, the broadband light absorption of porphyrin ligand, and strong photoelectronic properties derived from π-electron conjugation, this system achieved efficient photocatalytic and laccase-mimetic co-degradation of OTA in edible oils without the need for hydrogen peroxide (Figure 4a). To improve recoverability, PCN-222(Mn) was immobilized on edible fungal mycelial membranes. SEM revealed a dense biofilm structure with rough surfaces and protruding features, and elemental mapping confirmed the uniform distribution of Mn and Zr, collectively verifying successful composite membrane fabrication. In OTA-contaminated edible oils, PCN-222(Mn) achieved removal rates of 75.8% to 90.25% after 60 min of UV irradiation. When incorporated into the biofilm, the combined adsorption and molecular sieving capabilities of the mycelial matrix slightly enhanced degradation performance. Notably, the treated oils showed no significant changes in key physicochemical parameters.

For enhancing selective identification of target molecules in the adsorption–photocatalysis coupled systems, molecular recognition elements such as MIPs, aptamers, and antibodies, are incorporated [82,99,100]. This approach is expected to improve the efficiency of capturing and degrading mycotoxins in complex food matrices. Nisa et al. [101] developed a magnetic imprinted photocatalyst (Fe_3_O_4_@SiO_2_@TiO_2_@MIP) by sequentially depositing a TiO_2_ semiconductor layer onto silica-coated iron oxide nanoparticles, followed by MIP synthesis using coumarin (DMC) as a dummy template. TEM images revealed a progressive increase in particle size with each functionalized shell layer, culminating in a thick outer shell with evident agglomeration. Three-dimensional atomic force microscope (3D-AFM) analysis confirmed the successful modification of MIP.

The intermediate silica layer, a wide-bandgap nonconductor, effectively suppressed unfavorable heterojunction formation between Fe_3_O_4_ and TiO_2_. To optimize the capping layer, the following four configurations were evaluated for AFB_1_ photodegradation: Fe_3_O_4_@SiO_2_@TiO_2_@MIP, Fe_3_O_4_@SiO_2_@TiO_2_@NIP, Fe_3_O_4_@SiO_2_@MIP@TiO_2_, and Fe_3_O_4_@SiO_2_@NIP@TiO_2_. Several key parameters, including light source (UV/visible), UV intensity, irradiation duration (0–5 h), catalyst dosage (5–20 mg), and initial AFB_1_ concentration (50–200 μg/L), were systematically investigated. The optimal degradation conditions were found to be UV light, 45 W, 10 mg catalyst, 100 μg/L AFB_1_, and 5 h of irradiation. In spiked chili oil (12 μg/kg AFB_1_), Fe_3_O_4_@SiO_2_@TiO_2_@MIP achieved 95.51% removal within 50 min under UV light, with negligible degradation of capsaicin or ascorbic acid, confirming matrix selectivity. The catalyst retained about 100% efficiency over seven cycles without metal leaching, demonstrating exceptional stability for food safety applications.

#### 3.1.2. Visible-Light-Activated Systems

The adoption of milder visible-light irradiation, as opposed to UV-based excitation, meets the growing demand for more sustainable industrial processes, while significantly reducing damage to food matrices by eliminating the disruption of ROS produced by UV light [102]. From the perspective of catalyst design, various studies reported to enhance the excitation effect of visible-light, including exposing more active sites and enhancing the separation of charge carriers [103,104]. Thus, exploring novel photocatalysts that can leverage visible-light is of great importance.

Hu et al. [105] constructed a band-aligned heterojunction between graphitic carbon nitride (g-C_3_N_4_) and titanium dioxide (TiO_2_) to suppress charge carrier recombination under visible-light. The as-synthesized TiO_2_/SiO_2_ microspheres (TiSiMs) were coupled with tubular g-C_3_N_4_ (TCN) using a hydrothermal method for OTA removal in wine. Nitrogen adsorption–desorption isotherms revealed that the TiSiMs-TCN possessed a markedly increased specific surface area (53.4 m^2^/g) and well-defined mesoporosity (12 nm) compared with pristine g-C_3_N_4_ and SiO_2_, with this hierarchical pore architecture facilitating efficient toxin adsorption. Photoelectrochemical characterization confirmed superior optoelectronic properties, as follows: UV-Vis DRS and photoluminescence (PL) spectra revealed a narrowed bandgap (2.52 eV) and quenched PL intensity, indicating enhanced charge separation. Transient absorption spectroscopy showed a 3.2-fold increase in charge-carrier lifetime, attributable to the heterojunction and TCN tubular morphology, which together promoted electron transfer and improved light utilization. In real samples, OTA concentration was 100 μg/L under TiSiMs-TCN, close to the real contamination concentration, and the removal rate was more than 89.8% after 120 min of visible-light illumination. The safety of its intermediate products and final treated solution was verified by cellular activity and toxicology.

To address the difficulty in recycling photocatalyst in practical applications while effectively remediating toxins, a flexible PCL-g-C_3_N_4_/CQDs electrospun membrane was constructed by incorporating g-C_3_N_4_/CQDs heterojunctions into the membrane [106]. SEM and TEM characterizations confirmed the successful construction of the membrane. High-resolution XPS C 1s spectra identified graphitic carbon (C=C) from CQDs and sp^2^-hybridized carbon (N-C=N) in g-C_3_N_4_, indicating the linkage configuration of g-C_3_N_4_/CQDs within the membrane. The PCL-g-C_3_N_4_/CQDs electrospun membrane exhibited excellent activity in the removal of AFB_1_. The adsorption equilibrium was achieved within 30 min under dark conditions with 58.6% of AFB_1_ was adsorbed. Subsequently, AFB_1_ was degraded under visible-light irradiation. The membrane maintained above 96% AFB_1_ degradation efficiency across five consecutive cycles, showing good reusability.

Single-stranded nucleic acid ligands possess inherently high affinity for their complementary strands, enabling versatile target binding [107,108,109]. The programmability and conformational flexibility of aptamers endow them with enhanced stability during synthesis and superior nanomaterial-loading capacity [108]. Additionally, the structures and sequences of aptamer can be rationally designed in vitro to recognize target-specific characteristics, eliminating the need for conditional selection processes [100]. These advantages make aptamers more suitable than antibodies for real-matrix applications. To address the challenge of degrading trace aflatoxins (e.g., in peanut oil, typically <20 μg/kg), an aptamer-functionalized photocatalyst (MGO/TiO_2_-aptamer) was engineered by conjugating amine-rich AFB_1_ aptamers to carboxyl-activated magnetic graphene oxide-TiO_2_ composites [100]. This design boosted the AFB_1_ degradation rate constant from 0.0075 min^−1^ to 0.0096 min^−1^ (a 1.3-fold enhancement) in spiked peanut oil, displaying significant improvement in toxin capture and photocatalytic efficiency at low concentrations. Combined experimental and theoretical analyses elucidated the underlying mechanism. Frontier molecular orbital (FMO) calculations revealed that Ti/Fe incorporation reduced the bandgap of GO from 1.878 eV to 1.714 eV, with a further 0.028 eV decrease upon aptamer conjugation, enhancing UV–visible light excitation without compromising activity. The proposed degradation pathway involves aptamer-mediated AFB_1_ enrichment, followed by ·OH radical attack on the C8=C9 double bond via photogenerated holes and conduction band electrons (Figure 4b). This work establishes a paradigm for integrating aptamer recognition with photocatalytic degradation in food safety control.

Bio-based photocatalytic nanomaterials, created by integrating biomaterials with photocatalytic components, improve the safety of adsorption–photocatalysis coupled systems [110]. For example, Qiu et al. [111] linked graphitic carbon nitride to konjac glucomannan and introduced thiol (-SH) to fabricate a separation-free composite aerogel (g-C_3_N_4_-SH(Gl)@KG) that couples adsorption and photocatalysis for patulin (PAT) removal from apple juice. The -SH grafting treatment of graphite-phase carbon nitride (g-C_3_N_4_) increased the adsorption capacity of PAT and also facilitated photocatalytic efficiency. After five consecutive cycles, the aerogel remained 83% of its initial PAT adsorption capacity (0.92 mg/g). When applied to real apple juice samples, the adsorbent represented remarkable performance, achieving 53-fold and 46-fold higher adsorption capacities than unmodified g-C_3_N_4_ within 24 h and retaining 81% of its initial capacity after three adsorption–regeneration cycles. Moreover, no adverse effects on juice quality were observed.

### 3.2. Photothermal-Assisted Catalysis Systems

Thermally assisted adsorption–photocatalysis coupled systems represent a promising advancement in the field of photocatalysis, offering new avenues to enhance performance across a range of applications. The integration of materials such as plasmonic metal nanoparticles (e.g., Ag, Au) [112], narrow-bandgap semiconductors [113], transition metal compounds [114], and carbon-based materials into photocatalytic systems can significantly improve efficiency by harnessing photothermal effects [115]. By generating localized heat, these materials can promote enhanced charge-carrier separation [116], exciton generation, and reactant adsorption, boosting photocatalytic efficiency.

Lu et al. [117] explored the use of calcined nickel foam (NiO/Ni) as a substrate for immobilizing photocatalysts, effectively preventing their dispersion in solution. The modified Ni substrate not only exhibited excellent conductivity, adsorption capacity, and strong photothermal conversion. By loading recyclable ZnIn_2_S_4_ onto this substrate, an S-scheme heterojunction was constructed, achieving a remarkable H_2_ production rate of 92.3 μmol/h under light irradiation. This work provides valuable insights into the potential application of photothermal-assisted effects for detoxification in food.

Luo et al. [118] addressed challenges in copper sulfide (CuS) photocatalysis, such as rapid charge recombination and limited light absorption by integrating CuS with graphitic carbon nitride (g-C_3_N_4_) to fabircate a hybrid nanomaterial with dual photothermal and photocatalytic functionalities, along with the enrichment effect of g-C_3_N_4_. Their work on minimizing thermal losses and improving photothermal-assisted photocatalytic efficiency paves the way for environmental remediation. The work of Sun and his team further illustrated the dual functionality of photothermal and photocatalytic materials. Silver nanoparticles (Ag) possess intrinsic disinfection properties and localized surface plasmon resonance (LSPR) effects that enhance visible-light absorption and accelerate photogenerated charge separation, significantly improving the photocatalytic performance of composite materials [119]. By incorporating Ag-AgCl nanoparticles onto tetrahedral α-Fe_2_O_3_, they created a plasmonic composite that significantly improved photocatalytic disinfection performance, showing an impressive protection rate against *Aspergillus* spores. Through photoelectrochemical characterization, density functional theory calculations, and finite-difference time-domain simulations, the disinfection capability, electron sink behavior, and localized surface plasmon resonance effects of the photocatalyst were demonstrated. Under visible-light irradiation for 50 min, the protection rate against *Aspergillus* spores reached 93.65 ± 1.53%. Furthermore, the Ag-AgCl/α-Fe_2_O_3_ composite also exhibited high protective activity (90.52 ± 1.26%) during peanut storage. This work opens up avenues for food safety and antifungal applications, such as controlling fungal toxins in food storage.

These studies, particularly those exploring the application of thermally assisted photocatalysis in food safety, represent an exciting new frontier. With photocatalytic materials already showing promise in environmental remediation, further research into their use in food safety could revolutionize mycotoxin contamination control and prevention methods, contributing to public health and food security.

## 4. Adsorption–Biocatalysis Coupled Systems

Although current single-adsorption catalysts and adsorption–photocatalysis coupled systems have shown promising efficacy in mycotoxin detoxification, their practical implementation in the food industry remains hindered by several key challenges. These include ambiguous degradation pathways, potential nutrient loss, and excessive energy requirements [37]. In contrast, adsorption–biocatalysis coupled systems overcome these limitations by integrating enzymes that are capable of executing highly selective degradation [120]. Biocatalysis can mediate mycotoxins transformation via specific biochemical pathways and obtaining clearly structured conversion products [121]. Operating under mild conditions, enzymatic detoxification ensures both safety and sustainability. Therefore, the engineering of integrated platforms that couple enzymatic specificity with advanced adsorption materials offers a paradigm shift in mycotoxin management and presents considerable commercial potential for sustainable food safety solutions.

### 4.1. Enzyme for Mycotoxins Removal

Specific microbial-derived enzymes have been isolated, purified, and genetically engineered for the precise recognition and detoxification of mycotoxin, thereby transforming these hazardous compounds into benign or minimally toxic metabolites [120,121]. This biocatalytic decontamination strategy demonstrates unparalleled molecular precision, superior catalytic performance, and negligible secondary pollution. Given these distinctive merits, the direct implementation of these engineered enzymes to contaminated food matrices presents an ideal solution for mycotoxin mitigation, enabling comprehensive toxin breakdown without compromising the nutritional value of treated products. The currently common detoxification enzymes for mycotoxins mainly target toxins such as AFs, ZEN, DON, OTA, and PAT. However, these enzymes encounter problems such as low activity (requiring several days for the reaction) and poor stability, which limit their applications [22,57].

### 4.2. Adsorption–Enzymatic Catalysis Systems

The use of enzyme immobilization technology with adsorptive supports has become a significant advancement in the field of food detoxification, offering a promising alternative for improving the effectiveness of enzymes in food safety applications. By utilizing interfacial deposition, matrix entrapment, or covalent tethering methods, enzymes are immobilized onto engineered substrates, which improves both structural integrity and functional competence [57,122]. The resultant adsorption–enzymatic catalysis systems exploit the tailored physicochemical properties of supporting materials to reduce the enzyme–substrate spatial gap, dramatically enhancing mass transfer efficiency and overall catalytic performance while ensuring selective molecular recognition [123]. Furthermore, rational design enables these systems to establish specific reaction interfaces within multiphase food matrices, substantially broadening operational scope of enzymes [124]. Recent studies have validated the effectiveness of immobilized enzyme systems for removal of mycotoxins in food [52,125], highlighting the transformative potential of bifunctional adsorption–biocatalytic platforms for industrial implementation.

**Figure 5:**
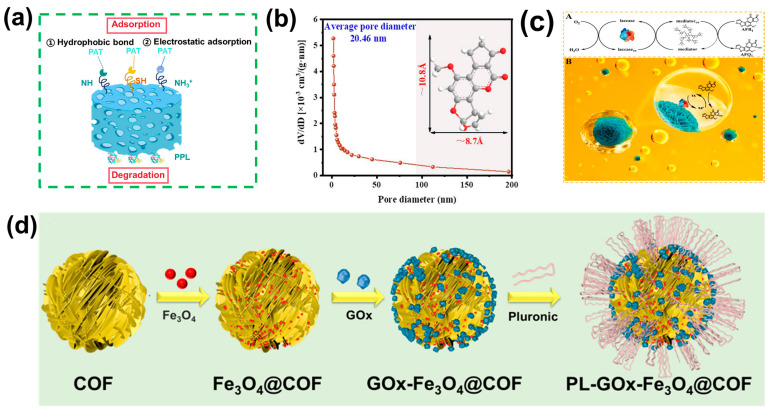
(**a**) Removal mechanism of PAT by CMC@HMMOF-Cys/PPL aerogel. (**b**) Pore-size distributions of Lac NF-P. (**c**) (**A**) Reaction mechanism of AFB_1_ oxidation catalyzed by the laccase–TA system. (**B**) Schematic illustration for the biodegradation of AFB_1_ in peanut oil catalyzed by Lac NF-P. (**b**,**c**) Reproduced with permission (Lu et al., 2023) [130] Copyright © 2023, American Chemical Society. (**d**) Preparation process of PL-GOx-Fe_3_O_4_@COF metal–biological hybrid catalyst. Reproduced with permission (Fu et al., 2024) [134] Copyright © 2024, American Chemical Society.

Porous adsorbents with hierarchical mesoporous structures, particularly COFs and MOFs, have emerged as promising matrices for enzyme immobilization due to their extraordinary specific surface areas and multifunctional surface chemistries [69,126]. To enhance reusability and facilitate the recovery of enzyme catalysts in complex food matrices, various strategies such as magnetic nanoparticle integration and hydrogel network fabrication have been developed. For example, Yan and coworkers co-immobilized cysteine and porcine pancreatic lipase into a hierarchical mesoporous zirconium MOF (CMC@HMMOF-Cys/PPL) [127]. The immobilized enzyme was then doped on an aerogel through the ex situ method and self-assembly strategy. This system proved highly effective in removing PAT from apple juice. The Zr-OH group on the HMMOFs interacted with the carboxyl group of biomolecules to achieve the co-immobilization of Cys and PPL without cross-linking reagents. This strategy demonstrated exceptional loading capacities, which were 16-fold and 4-fold higher than those of other carriers, respectively. The strong adsorption of HMMOFs, confirmed by BET, toward PAT was 38.41 μg/mg. The biodegradation of PPL was 28.68 μg/mg at 10 μg/mL PAT, attributable to the continuous increase in removal capacity over time. The synergistic effect of adsorption and degradation is demonstrated in Figure 5a. Additionally, excellent reusability, storage stability, and improved selectivity in simulated apple juice were also observed. To achieve continuous removal of PAT from real apple juice, CMC@HMMOF-Cys/PPL was subsequently filled into a continuous flow reactor. Moreover, the biologically derived components exhibited excellent biosafety profiles without significantly affecting the quality of apple juice.

In another example, polydopamine-coated membranes with reactive catechol and quinone groups were employed for enzyme immobilization. These groups facilitate spontaneous conjugation with thiol- or amine-containing biomolecules, enabling catalyst immobilization without exogenous coupling agents. An ultrasound-assisted polydopamine-functionalized magnetic porous chitosan (MPCTS@PDA@pancreatin) was designed for reducing OTA in wine [128]. Compared with the free enzyme, the detoxification rate of OTA was significantly enhanced, along with enhanced thermal stability (17–47 °C) and acid resistance (pH 3.0–7.0). In the presence of magnetic separation, MPCTS@PDA@pancreatin remained highly catalytic activity after eight cycles. Zhang et al. [129] developed a co-immobilization system by linking an aldo-keto reductase to a nanocarrier for degrading PAT in fresh pear juice. The nanocarrier was synthesized by imparting magnetite nanoparticles to cellulose nanocrystals (CNCs), and then functionalizing dopamine (DA) and polyethyleneimine (PEI) (DA/PEI@Fe_3_O_4_/CNCs). Using NADPH as a coenzyme, this system achieved a 98% removal of PAT in pear juice, with no impact on the quality of juice. In addition, due to the large surface area, high magnetization value, and oxygen/amine functionality, the stability and reusability of reductase were increased, with a detoxification rate over 87% after five cycles, retaining 62% of its activity after 14 days of storage at 4 °C. However, the use of coenzymes in food systems remains a problem to be solved in practical applications.

For removing mycotoxins from edible oils, Lu et al. recently developed an amphiphilic laccase–inorganic hybrid nanoflower (Lac NF-P) [130]. Laccase was immobilized within the inorganic nanoflowers (Lac NF) by the co-precipitation of copper sulfate pentahydrate and laccase-containing phosphate buffer. Subsequently, the amphiphilic polymer Pluronic F127 (PEO-PPO-PEO) was conjugated onto Lac NF using the cross-linking agent concanavalin A (ConA), endowing the hybrid system with exceptional dispersibility and stability in both aqueous and oil phases. With this operation, efficient toxin adsorption was facilitated while maintaining localized aqueous microenvironment around the enzyme to preserve its catalytic activity. Confocal microscopy images confirmed the distribution of Lac and ConA, while BET surface area and pore diameter displayed the expected minimal mass transfer resistance (Figure 5b). Furthermore, tannic acid (TA) served as a redox mediator, where its enzymatically oxidized cationic radicals further degraded AFB_1_, significantly raising the catalytic efficiency of laccase (Figure 5c). The results demonstrated that Lac NF-P exhibited a remarkable 134-fold and 3.2-fold increase in AFB_1_ degradation efficiency compared to free laccase and Lac NF, respectively. Notably, the treatment did not compromise peanut oil quality, and no catalyst leakage was detected. Toxicological analysis illustrated that the degradation product was almost non-toxic to human liver cells.

This work highlights that the rational design of amphiphilic immobilized enzyme catalysts can maintain the high activity of natural enzymes while enabling efficient mycotoxin removal in edible oils and dairy products. Furthermore, the suggested integration of a laccase–redox mediator system significantly improves the enzymatic degradation efficiency for AFB_1_.

Chen et al. [131] developed a zearalenone lactonase–inorganic hybrid nanoflower (InHNF-ZHD518) using a split intein moiety, enabling rapid and site-specific immobilization of ZHD518 directly from crude cell lysates without the need for organic solvents. The immobilized biocatalyst demonstrated a 40–60% increase in specific activity compared to the free enzyme, maintained structural stability across a wide pH range (3–11), and exhibited remarkable operational durability, retaining over 70% of its initial activity after eight reuse cycles. In practical ZEN detoxification assays using beer samples, InHNF-ZHD518 achieved more than 50% degradation of ZEN, whereas the free enzyme was mostly inactivated under the same conditions. The InHNF strategy combines several benefits, including environmentally friendly immobilization, purification-free processing, and enhanced catalytic performance, showcasing its strong potential for applications in food enzyme engineering and industrial biocatalysis.

### 4.3. Adsorption–Chemoenzymatic Catalysis Coupled Systems

Owing to the limited substrate spectrum available for enzymatic reactions, the integration of enzymatic catalysis with chemical catalysis to establish chemoenzymatic cascade systems exhibiting both high activity and stability has attracted increasing attention in recent years [120]. Through synergistic cascade reactions and colocalization strategies, spatial confinement effects and synergistic interactions can be achieved, thereby accelerating reaction kinetics [132,133]. However, research on the application of biochemical cascade reactions coupled with enrichment strategies for mycotoxin decontamination in food systems remains scarce.

A representative study in this area is the work by Fu et al. [134], in which GOx and Fe_3_O_4_ NPs were co-immobilized on an amphipathic covalent organic framework (COFs), forming a hybrid catalyst denoted as PL-GOx-Fe_3_O_4_@COF. During the synthesis process, iron ions (Fe^3+^) were in situ reduced within the pores of COF. GOx was subsequently immobilized on the surface through a Schiff base reaction (Figure 5d). This design enabled the separate yet confined loading of GOx and Fe NPs within one compartment, enhancing intermediate diffusion efficiency. Furthermore, an aldehyde-functionalized triblock copolymer was grafted to improve the mass transfer efficiency for applications in edible oils.

This hybrid catalyst successfully combined the Fenton reaction with enzymatic catalysis, achieving a metal–biological cascade system for mycotoxin removal. The targeted detoxification of mycotoxins can be realized due to the π-π stacking interaction between COFs and mycotoxins (especially AFB_1_), and convenient separation in edible oils. The flower-like morphology of the hybrid catalysts was characterized by TEM and fluorescence microscopy, which also confirmed the successful co-immobilization of enzymes and Fe_3_O_4_ NPs. Mechanistically, continuous hydrogen peroxide generation by GOx triggered the Fenton reaction, producing reactive oxygen radicals, while the gluconic acid formed in the oxidation process helped regulate the microenvironment, thereby stabilizing Fe^2+^/Fe^3+^ redox cycling. As a result, the cascade system achieved a detoxification efficiency against AFB_1_ that was six times higher than that of a simple mixture of free GOx and Fe_3_O_4_. Moreover, the catalytic activity was maintained after six reuse cycles without significant morphological changes. Importantly, no notable deterioration in the nutritional composition of peanut oil, and almost no toxicity of the degradation products and treated oil in human liver cells, was observed, underscoring the potential of this strategy for mycotoxin detoxification in vegetable oils.

## 5. Conclusions and Prospects

The adsorption–degradation coupled systems integrate the advantages of adsorptive support materials and catalytic components, enabling a seamless cascade of adsorption and degradation processes with high catalytic efficiency. Systems composed of molecular recognition materials and catalysts in the adsorption–photocatalyst system achieve directional mycotoxin detoxification in complex food matrices through synergistic interactions, while featuring facile synthesis and strong material specificity. This review summarizes enrichment-capable adsorbents used in the development of such bifunctional systems, along with their individual roles in mitigating fungal toxin contamination in food. Furthermore, the existing synergistic adsorption–photocatalytic and adsorption–biocatalytic systems were summarized for the first time, elucidating their design principles, bifunctional mechanisms, application performances, and potential toxicological aspects in the food matrices.

However, due to the complexity of food matrices, diversity of toxins, trace-level contamination, and stringent food safety standards, the available range of enrichment supports and catalysts for constructing systems that combine precision, efficiency, and biocompatibility remains limited. Future research is expected to focus on designing synergistic and directional adsorption–degradation systems and developing novel cascade reactions to enhance catalytic performance for efficient mycotoxin removal. These systems aim to eliminate trace and ultra-trace mycotoxins in food. Smart materials that are capable of conformational responses to temperature, pH, and other stimuli may regulate toxin-binding capacity, providing an ideal structure for adsorbents. In addition, technologies such as machine learning-assisted aptamer screening, may serve as essential tools in synthesizing the bifunctional system. Despite promising laboratory outcomes, the application of integrated enrichment–degradation materials in large-scale food processing remains limited. Thus, the development of non-toxic, cost-effective, and scalable industrial synthesis methods represents a key direction for future applied research, potentially advancing the practical implementation of these innovative adsorption–degradation coupled systems.

## Data Availability

No new data were created or analyzed in this study.
